# A Problem-Solving Therapy With Apple Watch Support for College Students With Alcohol Use Disorder Symptoms: Pilot Randomized Controlled Trial

**DOI:** 10.2196/88846

**Published:** 2026-10-05

**Authors:** Hagar Hallihan, Sangeun Lee, Ruth Adomah, Robert Miranda Jr, Kathleen Rospenda

**Affiliations:** 1 Department of Medicine, University of Illinois Chicago, 840 South Wood Street Clinical Science North Room 415, MC 718, Chicago, IL, 60612, United States, 1 312-413-5361; 2 School of Nursing, College of Health Professions and Sciences, University of Wisconsin–Milwaukee, Milwaukee, WI, United States; 3 Psychiatry & Human Behavior, Warren Alpert Medical School, Brown University, Providence, RI, United States; 4 Department of Psychiatry, University of Illinois Chicago, Chicago, IL, United States

**Keywords:** AUD, alcohol use disorder, feasibility study, digital health, college students

## Abstract

**Background:**

Alcohol use disorder (AUD) involves an impaired ability to stop or control alcohol use despite adverse consequences and represents a major public health problem. While AUD is most prevalent among college students, integrated evidence-based treatments for this population are lacking.

**Objective:**

The objectives of our study were to test the feasibility and acceptability of a newly developed behavioral intervention, problem-solving therapy and Apple Watch (PST-APPLE), and to preliminarily investigate the effectiveness of PST-APPLE among college students with symptoms of AUD.

**Methods:**

Participants were recruited through online advertising and on campus at universities in the Chicago area between May and September 2024. They were randomized in a 1:1 ratio using block randomization to the intervention group (PST-APPLE: n=12 individuals) or the control group (education-only: n=14 individuals), with the data analyst blinded to treatment assignment. Participants in the intervention group completed 12 weeks of the PST-APPLE intervention, delivered remotely via Zoom videoconferencing and interactions with the Apple Watch. Participants in the control group were asked to watch a 20-minute abstinence-motivation video and then participate in a 30-minute group discussion via Zoom. All participants completed follow-up assessments at 3 months. Outcomes included alcohol-related problems (Alcohol Use Disorders Identification Test [AUDIT] and Rutgers Alcohol Problem Index [RAPI]) and drinking motivation (Drinking Motives Questionnaire Revised [DMQR]).

**Results:**

We enrolled 26 participants aged 18‐25 years. The mean age was 22.5 (SD 2.20) years. We found reduced scores between preintervention and postintervention measures in the intervention group using 2-tailed paired *t* tests and baseline-adjusted analysis of covariance, including reduced alcohol misuse and problems (AUDIT and RAPI scores) and reduced DMQR social and coping motivation scores. Baseline-adjusted mean differences comparing the intervention with the control group showed reductions in alcohol use (AUDIT; mean difference −4.70, 95% CI −8.69 to −0.70) and in DMQR social motivation (mean difference −0.85, 95% CI −1.51 to −0.18) and enhancement motivation scores (mean difference −0.75, 95% CI −1.47 to −0.04).

**Conclusions:**

Our study findings indicate that the PST-APPLE intervention is feasible, acceptable, and hypothesis generating among college students with AUD. While findings are promising, this pilot study was not powered for definitive efficacy testing. A fully powered randomized controlled trial is needed to confirm these effects. Future studies could adapt PST-APPLE to other wrist-worn electronic devices/watches that capture physiological data (eg, Fitbit, Samsung Galaxy Watch) to increase reach and scalability, particularly among younger adults and digitally literate populations.

## Introduction

Alcohol use disorder (AUD) is a chronic disease characterized by an impaired ability to stop or control alcohol use, with craving and clinically significant impairment in occupational, social, or health functioning [1]. It is a major public health problem, and it is the most prevalent among college students (aged 18‐25 y) [1,2]. AUD contributes to more than 140,000 deaths and US $249 billion in costs annually in the United States [3,4]. About 49.6% of full-time college students aged 18 to 25 years drank alcohol in the past month [1]. Of those, about 29.3% engaged in risky drinking such as binge drinking [1], with 13% meeting the criteria for AUD. Heavy alcohol consumption impairs brain structures and cognitive functions like decision-making, problem-solving, and critical thinking, more severely in college-aged young adults [5,6]. The National Institute on Alcohol Abuse and Alcoholism estimates that approximately 1519 college students aged 18-24 years die each year from alcohol-related unintentional injuries, including motor vehicle crashes, and 696,000 students aged 18-24 years are assaulted by another student who has been drinking [1,2]. Because of the societal and individual costs associated with AUD among college students, reducing alcohol misuse in this population has been a target of intervention efforts for decades.

There are several evidence-based treatments for AUD in college students, such as motivational interviewing and personalized normative feedback (PNF) [7,8]. Motivational interviewing and PNF are among the most widely studied behavioral interventions for reducing hazardous alcohol use among college students [9-12]. Meta-analytic evidence suggests that these interventions can reduce alcohol consumption, binge drinking frequency, and alcohol-related harm in the short term [9,10]. However, intervention effects are often modest and may decrease over time, particularly when interventions are delivered as brief stand-alone approaches or when sustained engagement is limited [10,12]. PNF interventions aim to reduce hazardous drinking by correcting students’ misperceptions regarding peer alcohol consumption norms [11]. Although these approaches have demonstrated efficacy in reducing drinking quantity and frequency among college students, maintaining long-term behavior change and intervention engagement remains challenging in real-world settings characterized by dynamic social and environmental drinking cues [10-12].

Another evidence-based treatment that has shown particular promise for the treatment of AUD in young adults is problem-solving therapy (PST). PST is a brief psychotherapy treatment that has been recommended by the National Institute on Alcohol Abuse and Alcoholism and is recognized as an evidence-based practice by the American Psychological Association [3,4] for AUD, based on its proven efficacy in addressing the underlying issues that contribute to substance misuse across different age groups, including young adults [6,13,14]. PST is a form of “self-control training,” relevant to addressing risky behaviors such as heavy alcohol consumption, which may lead to the development of AUD. Our own systematic review [15], along with 3 randomized clinical trials of PST by other investigators in diverse populations, including young adults with AUD, showed that PST reduced alcohol use, but with small to moderate effect sizes (*d*=0.20-0.40) and with limited efficacy seen in young adults [16-18]. In addition, traditional PST delivery formats may have limited reach and engagement among college students, who often prefer technology-mediated, real-time, and personalized interventions [19].

However, existing single-modality evidence-based treatments for AUD, including PST, may have limitations in effectiveness or sustained engagement and may be more effective when combined with other treatment modalities. The effectiveness of integrated interventions has been explored in both adolescent and general adult populations [15,20]. For example, Belay et al [20] conducted a systematic review and meta-analysis evaluating psychosocial treatments for adolescents and young adults with AUD and found that integrated interventions combining multiple cognitive and behavioral approaches were associated with improved alcohol-related outcomes, including reductions in alcohol use frequency and consumption and greater abstinence rates at follow-up. Similarly, our previous systematic review of integrated behavioral interventions for adults with AUD demonstrated that integrated approaches produced better outcomes than single-modality interventions, including reductions in heavy drinking and alcohol consumption as well as improvements in mental health outcomes, such as depressive symptoms [15]. However, that review was limited to the general adult population. Together, these findings suggest that integrated interventions may offer advantages across age groups and clinical presentations, while also highlighting the need to evaluate their effectiveness in more specific and high-risk populations.

Digital health interventions, including mobile apps, wearable technologies, and web-based programs, have been increasingly explored to support alcohol reduction and self-management. Prior systematic reviews suggest that technology-assisted interventions can reduce alcohol consumption and improve engagement in care among individuals with risky alcohol use, including college students and young adults [21-23]. Technology-assisted approaches may be particularly relevant for college populations because they can support intervention delivery in naturalistic settings while reducing barriers related to stigma, scheduling, and treatment access.

Recent advances in wearable technology have demonstrated promise in delivering and monitoring behavioral health interventions among young adults [24,25]. Smartwatch-based interventions can enhance engagement, provide real-time physiological monitoring, and support behavior change, particularly among digitally native college students who exhibit high rates of wearable device ownership [24-26]. In parallel, emerging research using wearable sensor data and machine learning approaches has shown potential for identifying psychological states and health conditions in young adults [24,25], suggesting that physiological data from wearable devices may offer valuable real-time feedback to support behavioral interventions. The integration of wearable technology, such as the Apple Watch (Apple Inc), may therefore enhance PSTs accessibility, timeliness, and ecological validity by enabling continuous monitoring, real-time feedback, and intervention delivery in naturalistic settings. This capability supports just-in-time adaptive interventions that can provide support during moments of heightened risk [26]. For college students with AUD, such an approach may facilitate the delivery and reinforcement of PST strategies in real-world contexts where alcohol-related cues and decision-making challenges commonly occur. One question is whether the combination of PST and the Apple Watch may also affect motives for alcohol use. Drinking motives are a well-established predictor of alcohol use patterns and alcohol-related problems among college students [27-29]. Drinking motives include coping with stress, enhancing positive emotions, or facilitating social interaction [29]. These are considered crucial factors that affect an individual’s alcohol use and are associated with the development of AUD [30]. Prior research in US college student populations has shown that coping motives (ie, drinking to reduce negative emotions) and enhancement motives (ie, drinking to increase positive affect) are associated with heavier alcohol consumption, binge drinking, alcohol-related consequences, and greater risk for developing AUD symptoms [27-29,31]. In particular, coping-motivated drinking has been consistently linked to problematic alcohol use trajectories and increased psychological distress among emerging adults [28,31]. A longitudinal study further suggests that students who endorse coping-related drinking motives are more likely to experience persistent alcohol-related problems over time, supporting the role of drinking motives in the development and maintenance of AUD risk [31]. These findings suggest that drinking motives may represent an important mechanism underlying both alcohol misuse and responsiveness to behavioral interventions in college populations [23].

In the present study, we aim to evaluate the feasibility and acceptability of a novel integrated problem-solving therapy and Apple Watch (PST-APPLE) intervention targeting college students with AUD. In addition, the exploratory aim of this study is to examine whether this novel integrated intervention demonstrates preliminary effects in reducing indicators of AUD and drinking motives among college students. Since this is a pilot study and is not powered to definitively test efficacy, the findings will inform the design and power estimates of a future trial.

## Methods

### Study Design

A 2-arm randomized controlled trial was conducted to test PST-APPLE’s feasibility and acceptability in college students with symptoms of AUD and to gather preliminary data regarding the effectiveness of the intervention in reducing symptoms of AUD.

### Feasibility Test

We recruited participants through both online advertisements and in-person recruitment in the Chicago area at the University of Illinois at Chicago and Rush University. Participants were eligible if they were college students of any gender; were aged between 18 and 25 years; spoke English; currently used alcohol on 2 or more days per week as determined by Alcohol Use Disorders Identification Test (AUDIT) phone screening; met criteria for (past year) mild, moderate, or severe AUD during the Alcohol Use Disorder and Associated Disabilities Interview Schedule-5 (AUDADIS-5 interview); provided written informed consent; and were interested in cutting back alcohol intake or changing their drinking behaviors as assessed during the baseline screening study visit. Exclusion criteria included current participation in other alcohol treatment or use of medication (eg, naltrexone) in the past 6 months; diagnosis of severe mental illness (ie, schizophrenia, bipolar disorder, or any psychotic disorder); not currently enrolled in college; and pregnancy or intention to become pregnant.

Based on prior evidence, we assumed small to moderate effects on alcohol consumption [32]. A priori power calculation was conducted in G*Power (version 3.1.9) showing that the estimated required sample size was 44 participants to detect small (Cohen *f*=0.25) within-between interaction effects using repeated-measures ANOVA (α=.05, groups=2, repetitions =3, correlation=0.50). Based on this calculation, we aimed to recruit 44 participants for the final sample to provide preliminary effect size estimates for future adequately powered trials and to assess feasibility outcomes, including recruitment, retention, and intervention adherence.

Interested individuals completed an initial eligibility screening via a REDCap (Vanderbilt University) survey. Those who met preliminary criteria were invited to attend a tele-orientation session and complete a baseline assessment. Participants were recruited from May to September 2024. We initially screened 132 participants. Of these, 106 were excluded because they did not meet eligibility criteria or declined to participate due to busy schedules. The remaining 26 participants were formally enrolled in the study, completed all study procedures, and were included in the final data analysis.

### Randomization

To minimize selection bias and ensure treatment group balance, block randomization was used to assign participants to the PST-APPLE intervention or education-only control group in a 1:1 ratio. The randomization sequence was generated in Excel by a research team member using a fixed block size of 4, with 2 assignments to each study group within each block, and was embedded within the REDCap randomization system. Following eligibility screening and completion of baseline assessments, participants were enrolled by trained research staff and assigned to study groups via REDCap. Outcome data were collected by participants via REDCap. The data analyst was blinded to participant treatment assignment until the primary data lock to reduce bias during outcome analysis. Due to the nature of the intervention, participants and the counselor could not be blinded to treatment allocation.

### Intervention Group: PST-APPLE Intervention

Participants in the PST-APPLE intervention group completed 12 weeks of the PST-APPLE intervention, delivered remotely via Zoom (Zoom Communications) videoconferences and interactions with an Apple Watch. During weeks 1 to 6, the first 6 sessions were provided as one-on-one sessions, which were delivered weekly via Zoom by a counselor. Each participant met once a week for 45‐60 minutes and engaged in PST with a counselor while simultaneously interacting with an Apple Watch. Additionally, participants reviewed prediscussed Apple Watch data (ie, heart rate [HR], sleep patterns, and physical activity such as step counts) together with the counselor during weekly Zoom sessions. The PST is a structured intervention session, and its components include problem identification, goal setting, solution discussions, and action planning.

In weeks 7 to 12, the remaining 6 sessions were conducted as individual sessions, using only the Apple Watch. In addition to the standardized Apple Watch notification prompts developed by the research team to support engagement with the intervention, participants received Apple Watch’s built-in automated notifications (eg, activity completion pop-ups, activity ring closure, and activity achievement badges), which provided visual encouragement and reinforced progress toward participants’ personal goals. Furthermore, participants received brief weekly check-in reminders to apply problem-solving steps to real-life situations, self-monitored their stress levels through HR data, and reviewed their progress on sleep and physical activity (eg, daily step counts) goals established during the counselor-led sessions. The counselor in the intervention sessions was a registered nurse who held a master’s degree in Nursing Health Science and Divinity. All counselor-led PST sessions were recorded to facilitate intervention monitoring and quality assurance. The counselor participated in regular supervision meetings with the principal investigator (PI) to review intervention delivery and participant progress. A comprehensive description of the intervention is available in the supplementary protocol (Multimedia Appendix 1), which is reported in accordance with the CONSORT (Consolidated Standards of Reporting Trials) extension for pilot and feasibility trials (Checklist 1) and the TIDieR (Template for Intervention Description and Replication) checklist (Checklist 2) to facilitate transparency [33,34].

This intervention was designed for the broader young-adult population; however, the present sample consists of college students who were motivated to reduce or modify their drinking behavior.

### Protocol

Each participant in the intervention group was issued an Apple Watch and instructed to pair it with their personal phone. Participants were also required to pair their Apple Watches with the PI’s ABSTAIN LAB Apple Team account, which facilitated our team’s secure access to their wellness data. That pairing ensured that only relevant health data (fitness activity, HR, and sleep metrics) were accessible to the research team. The collected data were used to track participant adherence to the PST-APPLE intervention and to provide personalized feedback to participants regarding their progress on daily health goals. Specifically, participants received real-time feedback on: (1) fitness activity (daily walking step count, with a goal of 7000‐10,000 steps, important as regular exercise enhances cognitive flexibility and working memory) [35]; (2) HR monitoring (with alerts when HR indicates elevated stress, which can mitigate stress-induced impairments in executive function) [36]; and (3) sleep patterns (with a goal of 7‐9 hours per night, as sufficient sleep is associated with better cognitive function and attention regulation) [37]. These physiological markers were reviewed with the counselor during weeks 1 to 6 and used to set personalized goals for behavior change.

### Control Group: Education-Only Control

Participants in the control group were asked to watch “Quit Drinking Motivation,” a 20-minute video created by MotivationHub [38], and then participate in a 30-minute group discussion via Zoom. The discussion centered on their individual encounters with alcohol, the ways in which alcohol has influenced their lives, the repercussions stemming from alcohol consumption, and the insights gained from the “Quit Drinking Motivation” video. They were also asked to explore how these lessons from the video will shape their future decisions and actions. This approach allowed participants to receive basic alcohol-related educational content about health risks and to engage in discussion about alcohol use. The research team selected this comparison group because this pilot study was designed to evaluate the PST-APPLE intervention as a whole and was not intended to separate the independent effects of PST counseling or the Apple Watch component.

### Measures

#### Screening and Diagnostic Measures

For eligibility screening purposes, AUDIT was used to determine whether participants met the alcohol-related eligibility criterion. This 10-item self-reported instrument is considered a gold standard screening instrument for problematic drinking in young adults, including college students [39,40]. AUDADIS-5 is a structured diagnostic interview that contains *Diagnostic and Statistical Manual of Mental Disorders *(*Fifth Edition*; *DSM-5*) criteria for AUD diagnosis and severity [41]. The AUDADIS-5 was used only before enrollment and randomization to assess the presence of diagnostic criteria for AUD and classify the level of any existing AUD symptoms as mild, moderate, or severe.

#### Baseline Measures

After tele-orientation and informed consent were obtained, participants were sent a link to REDCap and independently completed the baseline questionnaires. The following were measured at the baseline time point (0 mo). AUDIT was used to determine participants’ symptoms of alcohol misuse before the intervention [39]. The Rutgers Alcohol Problem Index (RAPI) is an 18-item self-administered measure and was used to assess participants’ problematic drinking and negative consequences associated with alcohol use over the past year [42]. The Drinking Motives Questionnaire Revised (DMQR) is a 20-item self-administered measure and was used to assess participants’ motivation to drink alcohol, and it yields four domains, including social, coping, enhancement, and conformity [27]. These domains assess social motives, coping motives, enhancement motives, and external social pressure toward alcohol use, respectively.

#### Outcome Measures

For study outcomes, participants completed a postintervention survey, including the AUDIT, RAPI, and DMQR. At the end of the intervention, participants were sent a REDCap link as soon as possible and they independently completed the postintervention questionnaires. The questionnaires were completed within 1 week of the intervention. All outcome measures were self-reported questionnaires completed independently by participants via REDCap to minimize assessor bias.

#### Intervention Feasibility

Feasibility was assessed by participant retention throughout the study by monitoring the proportion of the sample formally enrolled and retained at 3-months.

#### Intervention Adherence

Participant adherence to the PST-APPLE intervention was assessed based on participant engagement, as evidenced by documented attendance at all 6 individual PST sessions, which were delivered weekly via Zoom by a counselor during the first 6 weeks while participants simultaneously interacted with the Apple Watch. For weeks 7-12, adherence was evaluated through the completion of 6 additional self-guided individual sessions conducted independently using only the Apple Watch.

#### Intervention Acceptability

To evaluate the study’s acceptability, the Client Counseling Satisfaction Scale, a 6-item 5-point Likert scale, was used to assess satisfaction with the intervention for participants in the intervention group [43].

### Data Analysis

All statistical analyses were performed using SPSS 29.0, (version 29; IBM Corp) and statistical significance was set at *P*<.05 (2-tailed). Prior to data analysis, normality testing was conducted using Shapiro-Wilk tests and Q-Q plots. Given the small sample size and the pilot nature and exploratory objectives of this study, no formal correction for multiple comparisons was applied; however, we acknowledge that testing multiple outcomes increases the risk of type I error. The primary aim was to examine preliminary intervention effects rather than to test efficacy. Therefore, all findings should be interpreted with caution and considered exploratory and hypothesis-generating rather than confirmatory. Descriptive statistics were used to summarize participants’ sociodemographic characteristics and the levels of outcome variables, such as AUDIT, RAPI, DMQR, and Client Counseling Satisfaction Scale. Two-tailed paired *t* tests were conducted to examine changes in outcome variables. The analysis of covariance (ANCOVA) was used to examine the effect of the intervention. Effect sizes are reported as Hedge g for within-group 2-tailed paired *t* tests (small=0.2, medium=0.5 and large=0.8) and partial η² for between-group ANCOVA analyses (small=0.01, medium=0.06, and large=0.14), following Cohen conventions. While different metrics are used due to different analysis types, both indicate meaningful effect sizes for primary outcomes. As baseline values were higher in the PST-APPLE group, we prioritized baseline-adjusted between-group analyses using ANCOVA as the primary analytic approach, controlling for baseline values in the main variables, such as AUDIT and DMQR scores. For each ANCOVA, the corresponding baseline was included as a covariate (eg, baseline AUDIT for postintervention AUDIT, baseline RAPI for postintervention RAPI, and baseline DMQR for postintervention DMQR). ANCOVA was used as a baseline-adjusted analytic approach to account for baseline group differences observed in this pilot study. Within-group changes were also examined using 2-tailed paired *t* tests. From weeks 7 to 12, adherence was assessed through completion of self-guided sessions using the Apple Watch, defined as responding to at least 70% of prompted tasks per week. These tasks included applying problem-solving steps to real-life situations, monitoring stress levels via HR data, and engaging with and making progress toward sleep and physical activity goals established during counselor-led sessions.

### Ethical Consideration

The Institutional Review Board for the University of Illinois at Chicago approved this study (STUDY2024-0268). All participants provided written informed consent prior to participation and agreed to participate in the study voluntarily. Participant data were collected and managed using secure, password-protected systems (ie, REDCap), and all data were deidentified to protect participant privacy and confidentiality. Participants in both groups were compensated with an Apple Watch upon completion of study procedures.

## Results

Figure 1 shows the CONSORT flow diagram of participant enrollment and follow-up through data analysis. This shows a 100% retention rate among enrolled participants (n=26; see Figure 1).

**Figure 1.**
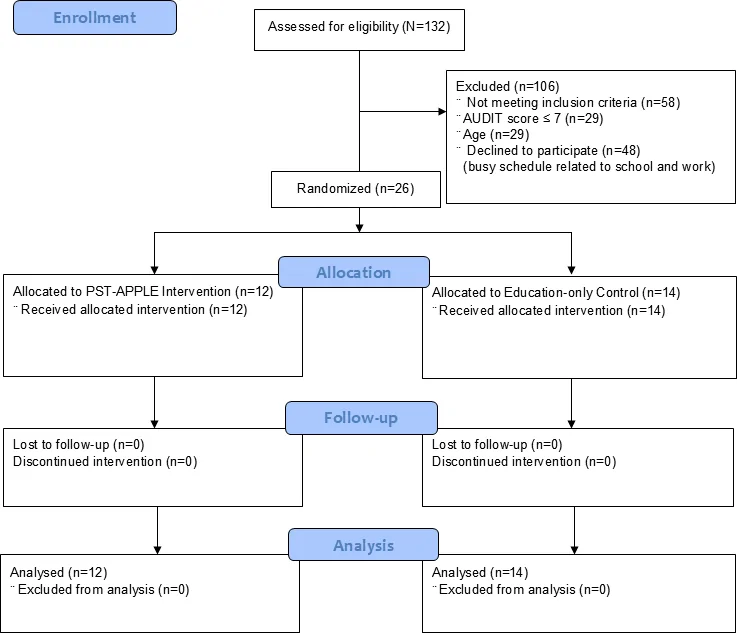
CONSORT (Consolidated Standards of Reporting Trials) flow diagram showing enrollment and follow-up to the data analysis. AUDIT: Alcohol Use Disorders Identification Test; PST-APPLE: problem-solving therapy and APPLE Watch.

### Sample Characteristics

Baseline characteristics are presented in Table 1. Although randomization was performed, the PST-APPLE group showed higher baseline values on the primary outcome measures (AUDIT and RAPI), likely due to chance given the small sample size. The mean age of participants was 22.5 (SD 2.20, range 18‐25) years, and 50% (13/26) of the participants were female. Regarding race, 65.4% (17/26) were Asian, followed by Hispanic (4/26, 15.4%), White (3/26, 11.5%), and African American (2/26, 7.7%). After randomization, 12 participants were assigned to PST-APPLE, and 14 were assigned to the education-only control group.

**Table 1.** Sample characteristics.

Variables		Overall sample (N=26)	PST-APPLE^a^ group (n=12)	Education-only group (n=14)
Age (y), mean (SD; range)		22.54 (2.20; 18-25)	—^b^	—
Gender, n (%)				
Men		13 (50)	7 (58.3)	6 (42.9)
Women		13 (50)	5 (41.7)	8 (57.1)
Race/ethnicity, n (%)				
Non-Hispanic White		3 (11.5)	0 (0.0)	3 (21.4)
Hispanic		4 (15.4)	2 (16.7)	2 (14.3)
African American		2 (7.7)	0 (0.0)	2 (14.3)
Asian		17 (65.4)	10 (83.3)	7 (50.0)

a PST-APPLE: problem-solving therapy and Apple Watch.

b Not available.

### Differences in Outcomes Between the Intervention and Control Groups

Within the PST-APPLE group, scores decreased in AUDIT (mean difference 7.00, 95% CI 2.48-11.52, Hedge *g*=0.916), RAPI (mean difference 12.92, 95% CI 2.53-23.30, Hedge *g*=0.735), and reduced drinking motivation (DMQR social, mean difference 0.78, 95% CI 0.02-1.55, Hedge *g*=0.606; and DMQR coping, mean difference 0.60, 95% CI 0.02-1.18, Hedge *g*=0.613), representing medium to large effects in these outcomes as shown by Hedges *g*, which provides a small-sample size correction to Cohen *d*. Control group scores remained largely unchanged from baseline to follow-up scores for any outcome variable (Table 2).

ANCOVA results controlling for baseline values showed adjusted mean differences between the PST-APPLE vs education-only control groups: AUDIT −4.70, 95% CI −8.69 to −0.70; DMQR social −0.85, 95% CI −1.51 to −0.18; DMQR enhancement −0.75, 95% CI −1.47 to −0.04; DMQR coping −0.53, 95% CI −1.14 to 0.09; DMQR conformity −0.29, 95% CI −0.79 to 0.20; and RAPI −7.6, 95% CI −15.76 to 0.56 (Table 3). Negative adjusted mean differences indicate lower postintervention scores in the PST-APPLE group compared with the education-only control group.

**Table 2.** Within-Group pre and postintervention means and effect sizes.

Measure and group		Baseline mean (SD)	Postintervention mean (SD)	Mean difference (95% CI)	Hedge *g*
AUDIT^a^					
PST-APPLE^b^		15.75 (5.74)	8.75 (4.92)	7.00 (2.48 to 11.52)	0.916
Education-only		10.93 (5.69)	11.00 (5.55)	–0.07 (–1.66 to 1.52)	–0.024
DMQR^c^ social					
PST-APPLE		3.65 (1.02)	2.87 (0.83)	0.78 (0.02 to 1.55)	0.606
Education-only		3.71 (0.74)	3.74 (0.93)	–0.03 (–0.40 to 0.34)	–0.042
DMQR enhancement					
PST-APPLE		3.10 (1.25)	2.37 (0.91)	0.73 (–0.26 to 1.73)	0.435
Education-only		2.86 (1.07)	3.03 (1.00)	–0.17 (–0.44 to 0.10)	–0.343
DMQR coping					
PST-APPLE		2.93 (1.37)	2.33 (0.83)	0.60 (0.02 to 1.18)	0.613
Education-only		2.24 (0.97)	2.43 (1.16)	–0.19 (–0.63 to 0.26)	–0.229
DMQR conformity					
PST-APPLE		2.17 (0.87)	1.82 (0.62)	0.35 (–0.22 to 0.92)	0.365
Education-only		2.11 (0.74)	2.09 (0.76)	0.03 (–0.30 to 0.36)	0.048
RAPI^d^					
PST-APPLE		22.92 (14.78)	10.00 (6.76)	12.92 (2.53 to 23.30)	0.735
Education-only		11.79 (10.76)	13.43 (12.29)	–1.64 (–5.24 to 1.96)	–0.248

a AUDIT: Alcohol Use Disorders Identification Test.

b PST-APPLE: problem-solving therapy and Apple Watch.

c DMQR: Drinking Motives Questionnaire Revised.

d RAPI: Rutgers Alcohol Problem Index.

**Table 3.** ANCOVA^a^ results comparing Postintervention outcomes between groups.

Measure	Adjusted mean difference^b^ (95% CI)	*F* test (*df*)	*P* value	Effect size (partial η^2^)
AUDIT^c^	−4.70 (−8.69 to −0.70)	5.92 (1, 23)	.02	0.205
DMQR^d^ social	−0.85 (−1.51 to −0.18)	6.95 (1, 23)	.02	0.232
DMQR enhancement	−0.75 (−1.47 to −0.04)	4.77 (1, 23)	.04	0.172
DMQR coping	−0.53 (−1.14 to 0.09)	3.13 (1, 23)	.09	0.120
DMQR conformity	−0.29 (−0.79 to 0.20)	1.53 (1, 23)	.23	0.062
RAPI^e^	−7.6 (−15.76 to 0.56)	3.72 (1, 23)	.07	0.139

a ANCOVA: analysis of covariance.

b Adjusted mean difference (problem-solving therapy and Apple Watch, PST-APPLE–Control); negative values indicate lower scores in the PST-APPLE group.

c AUDIT: Alcohol Use Disorders Identification Test.

d DMQR: Drinking Motives Questionnaire Revised.

e RAPI: Rutgers Alcohol Problem Index.

### Feasibility, Acceptability, and Adherence of the Intervention

After randomization, all formally enrolled participants completed all study procedures, resulting in a 100% retention rate among enrolled participants. Participants in the intervention group indicated high acceptability of the intervention sessions (mean 4.38, SD 1.05) compared with the education-only control group (mean 3.85, SD 1.09), indicating that both the sessions and the counselor were helpful. Participants’ and the counselor’s evaluations of the acceptability and feasibility of the integrated PST-APPLE intervention were highly positive. All enrolled participants completed the intervention as designed. Adherence to the PST-APPLE intervention was confirmed by documented attendance at all 6 individual PST sessions delivered weekly via Zoom by a counselor during the first 6 weeks (100% session attendance), with concurrent interaction with the Apple Watch. During weeks 7 to 12, 91.7% (11/12) of participants completed at least 70% of prompted tasks per week. Apple Watch data further showed participants wore the device an average of 6.2 (SD 0.8) days per week and engaged with prompted tasks at an overall rate of 82%.

## Discussion

### Main Findings

In this study, we evaluated the feasibility and acceptability of a novel integrated PST-APPLE intervention targeting young adult college students with AUD. We also explored whether this novel integrated intervention showed promise in reducing indicators of AUD and drinking motives among college students. The newly developed intervention was successfully offered to college students with AUD who wanted to mitigate their problematic drinking behaviors.

Participants in the PST-APPLE group showed a reduction in AUD symptoms, including reduced levels of alcohol use problems (AUDIT and RAPI) and drinking motivation (DMQR social and coping). The reduction in AUDIT scores postintervention represents approximately a 3 to 4 point decrease. Although this finding is encouraging, it should be interpreted with caution given the pilot nature of the study and the small sample size. The wide confidence interval reflects the small sample size and suggests the need for larger trials to establish precise effect estimates. Given that AUDIT is a well-validated screening tool for hazardous and harmful drinking [39], these findings suggest that PST-APPLE may contribute meaningfully to public health efforts targeting alcohol misuse. These findings suggest improved effect sizes compared to prior psychosocial intervention studies for young students with AUD [15,20]. Our systematic review found PST effect sizes of *d*=0.20-0.40 for alcohol reduction [15], while the current intervention demonstrated large within-group effects. This improvement may be attributable to the integration of wearable technology (ie, Apple Watch) providing real-time physiological feedback and sustained engagement beyond traditional counselor contact [25].

Furthermore, the reduction in social and enhancement drinking motives is particularly noteworthy. As prior research shows, these motives are strong predictors of binge drinking and risky alcohol-related behaviors among young adults [29]. Thus, the observed decreases may reflect shifts not only in behavior but also in underlying cognitive and emotional patterns related to drinking. The reduction in social and enhancement drinking motives further suggests that PST-APPLE may not only reduce drinking behaviors but also alter underlying psychological drivers of alcohol use, such as using alcohol to cope with stress or facilitate social interaction. This is critical, as coping-related drinking motives have been linked with more severe and persistent forms of AUD [29,44]. By addressing these motives directly, PST-APPLE may help disrupt the cognitive-emotional pathways that perpetuate problematic drinking.

Given the pilot nature of this study, the small sample size, and the baseline differences between groups, the findings should be interpreted with caution. To better determine the effects of the intervention, future research with a larger sample is needed.

The mechanisms underlying PST-APPLE’s effectiveness warrant discussion. Beyond traditional PST components, the Apple Watch integration may have contributed through several pathways: (1) continuous self-monitoring of physiological stress markers (eg, HR) enabling real-time awareness and the application of coping strategies; (2) objective sleep and activity tracking reinforcing the connection between health behaviors and cognitive function; (3) automated reminders and prompts extending therapeutic contact beyond weekly sessions; and (4) gamification elements inherent in wearable fitness tracking potentially increasing motivation and engagement. Recent work has demonstrated that wearable sensor data can reliably detect psychological states, including anxiety, depression, and energy levels [23,24], supporting the theoretical basis that physiological monitoring may enhance awareness of AUD-related patterns and the application of problem-solving.

### Feasibility, Acceptability, and Adherence

The intervention’s feasibility, acceptability, and adherence were demonstrated by 100% enrollment and retention rates. This pattern aligns with previous studies suggesting that digital health interventions, especially when integrated with wearable technology (eg, the Apple Watch), can improve user engagement and intervention fidelity [45].

### Limitations

While PST-APPLE effectively addresses alcohol use and drinking motives, it is important to interpret our study findings in light of specific limitations. First, a key important limitation is the difference in contact time and attention between groups. The intervention group received 12 weeks of structured intervention (6 counselor sessions plus 6 Watch-based sessions), whereas the control group received a single 50-minute session. This raises the possibility that observed effects may partly reflect nonspecific factors such as therapeutic attention, rather than PST-APPLE–specific mechanisms. Future studies should employ attention-matched control conditions to isolate specific intervention effects.

Next, the small sample size (n=26) and recruitment from only 2 universities and online advertisements may limit the generalizability of the findings. Larger samples are needed in future studies to confirm these effects in broader populations. Since recruitment was more challenging than anticipated, the final sample size (n=26) was below the planned target of 44 participants. As a result, intervention effects should be interpreted with caution, and some nonsignificant findings may reflect limited statistical power. In addition, because this study focused on college students, the findings may not be generalizable to noncollege young adults aged 18‐25 years. Furthermore, selection bias may have occurred, as 7 of 33 (21%) initially recruited participants opted not to proceed during the tele-orientation phase. Those who enrolled may represent a more motivated or less severe subset of college students with AUD, potentially limiting generalizability to the broader population of students with alcohol problems. Future studies should collect data on nonenrollees to characterize this potential bias.

Also, 65% (17/26) of the participants identified as Asian, which limits the generalizability of the results to other racial/ethnic groups. Although baseline imbalances were statistically controlled for, they could still introduce biases not fully addressed by ANCOVA. Furthermore, this study assessed outcomes of 3-months postintervention. Future studies should consider extended follow-up to provide more information about the lasting impacts of the intervention on reduced alcohol use and changes in drinking motives.

Furthermore, this was a pilot feasibility study, and the intervention duration (ie, 60 min/session, 12 wk) may not reflect the optimal dosing required to achieve maximal effects. Future studies should examine the impact of different intervention durations and intensities.

Finally, we acknowledge that the control condition included significantly less contact time than the intervention group, which is a limitation of this pilot study. Future studies should consider an attention-matched control condition to better isolate the specific effects of PST-APPLE components.

Finally, although validated tools such as the AUDIT and RAPI were used, self-reported alcohol consumption is subject to social desirability and recall biases [46]. Future studies should consider using objective measures to strengthen the study’s findings.

### Implications for Future Research

Nonetheless, our study findings indicate that the PST-APPLE intervention is feasible, acceptable, and may be beneficial among college students with AUD. High adherence was related to several factors, including the use of wearable devices (ie, Apple Watch), weekly check-ins from the research team, and structured PST intervention sessions. Additionally, we reported effect sizes (Hedges *g* and η²), allowing for the assessment of the practical and clinical significance of the findings. Reporting effect sizes highlights the magnitude of change, which is crucial considering that this is intervention research. Furthermore, the focus on reducing social and coping drinking motives is an important strength, as these are known risk factors for persistent and problematic drinking [44]. By targeting motivational pathways, the PST-APPLE intervention may have long-term benefits that extend beyond reductions in alcohol consumption. Finally, the use of well-validated assessment tools such as the AUDIT [39], RAPI [42], and DMQR [27], strengthens the credibility of the findings. These tools have strong psychometric properties and are widely recognized in the field of substance use research [47,48].

Future research priorities include (1) conducting an adequately powered confirmatory randomized clinical trial with attention-matched control conditions to establish efficacy; (2) implementing longer follow-up periods (6‐12 mo) to assess durability of effects and the need for booster sessions; (3) conducting mechanistic studies using objective alcohol consumption biomarkers, ecological momentary assessment, and neurocognitive assessments to understand how PST-APPLE affects executive function and drinking behavior; (4) translating PST-APPLE to other wearable devices (such as Fitbit and Samsung Galaxy Watch) to increase accessibility; and (5) examining moderators of treatment response to identify which subgroups of college students benefit most from this approach. This would help determine whether the reductions in AUD symptoms and drinking motives are sustained or whether booster sessions are needed.

## Supplementary material

10.2196/88846 Multimedia Appendix 1 CONSORT TIDieR supplementary protocol.

10.2196/88846 Checklist 1 CONSORT checklist.

10.2196/88846 Checklist 2 TIDieR checklist.

## Abbreviations

ANCOVA: analysis of covariance
AUD: alcohol use disorder
AUDADIS-5: Alcohol Use Disorder and Associated Disabilities Interview Schedule-5
AUDIT: Alcohol Use Disorders Identification Test
CONSORT: Consolidated Standards of Reporting Trials
DMQR: Drinking Motives Questionnaire Revised
*DSM-5*: *Diagnostic and Statistical Manual of Mental Disorders* (*Fifth Edition*)
HR: heart rate
PI: principal investigator
PNF: personalized normative feedback
PST: problem-solving therapy
PST-APPLE: problem-solving therapy and Apple Watch
RAPI: Rutgers Alcohol Problem Index
TIDieR: Template for Intervention Description and Replication
