# Supplementary material for: A Problem-Solving Therapy With Apple Watch Support for College Students With Alcohol Use Disorder Symptoms: Pilot Randomized Controlled Trial
Source: JMIR Form Res. 2026 Oct 5;10:e88846. doi: 10.2196/88846 (PMC13637571; doi:10.2196/88846)
Supplement: Supplementary file 1 [file formative-v10-e88846-s001.docx]

**CONSORT TIDieR Supplementary File 1**

Session information

| Item | Description |
| --- | --- |
| Name | PST- APPLE intervention |
| Objectives | This integrated intervention helps participants manage their stress with coping skills and regulate negative emotions and thoughts by combining the structured problem-solving methods and the use of Apple watch self-monitoring approach when facing life stressors that contribute to alcohol use. |
| Materials | - Participant (digital booklet – sent via encrypted email): Intervention overview, Problem list, PST Worksheets, and an activity ideas handout for pleasant, social, and physical activities. - Provider: a standardized session-by-session counselor leader’s guide and an intervention tracking form (REDCap) - Devices/platforms: Zoom, Apple watch, and REDCap for data capture and tracking. - Availability: The full counselor leader’s guide is available from the corresponding author on reasonable request, and a more detailed description of intervention content is reported separately in Methods section. |
| Activities | - Problem solving: The counselor and participant collaboratively generate a Problem List, then work participant-selected problems through a structured seven-step process on a PST Worksheet, setting SMART (specific, measurable, actionable, realistic, time-bound) goals and action plans. - Activity planning: Each week the participant plans and self-monitors three activity types (physical, social, pleasant) intended to displace drinking. - Typical session flow: Rapport; Brief restatement of the PST model and its guiding principles; Review of the previous worksheet and home practice; Completion of problem solving and activity planning for the current week; Assignment of home activities and self-monitoring. - Apple Watch self-monitoring: Participants received feedback on daily steps, heart rate, and sleep. During weeks 1-6, data was reviewed with the counselor and used to set personalized health-behavior goals. During weeks 7-12, participants self-monitored the health data. - Engagement support: The research team developed a series of brief, standardized Apple Watch notification prompts to support engagement with the intervention. Notifications were delivered once daily between 7:00 AM and 10:00 AM and reminded participants to monitor their heart rate, aim for the recommended 7–9 hours of sleep, and participate in regular physical activity. The sample Apple Watch prompts were as follows (including behavioral targets): - *Heart rate monitoring*: “Take a moment to check your heart rate and stay aware of your progress today.” - *Sleep*: “Aim for 7–9 hours of sleep tonight to support your health and recovery*.”* - *Physical activity*: *“*A short walk or workout today helps you stay on track with your fitness goals*.*”   In addition, participants received automated notifications when they completed their personal health goals. These Apple Watch’s automated notifications were standard and device-native features. Furthermore, the counselor manually sent brief weekly check-in messages during weeks 7-12 to maintain engagement. |
| Provider information | Each intervention session was delivered by a study counselor who is a registered nurse holding a Master’s degree in Nursing Health Science and a Master of Divinity. The counselor helped guide participants throughout the protocol.  Before delivering the intervention, the counselor completed structured training in PST. Training comprised (1) PST model and its principles; (2) the seven-step problem-solving procedure; (3) SMART goal setting and activity planning; (4) review of the standardized counselor leader’s guide and participant materials; and (5) orientation to the Apple watch self-monitoring and to the REDCap.  The counselor conducted all research activities based on the manualized leader’s guide and audio-recorded all counselor-led sessions for monitoring and quality assurance. In addition, the counselor had regular supervision meetings with the principal investigator to review intervention delivery and participant progress. |
| Intervention description | The intervention was delivered individually for 12 weeks. During weeks 1-6, a counselor-led PST session was delivered via Zoom once per week. Each session lasted 45-60 minutes. Participants joined the sessions remotely using their computer. During weeks 7-12, the intervention was conducted based on self-directed form with the Apple Watch. In addition to the daily Apple Watch Prompts, a brief weekly check-in message was sent manually by the research team members (e.g., counselor and/or coordinator) to help participants apply problem-solving steps and review health-behavior goals independently.  Session content was structured but tailored in several ways. For example, participants selected the type of problems from their problem list. The counselor collaborated with participants to chose appropriate solutions and action plans to meet participant-specific SMART goals and introduced personalized health-behavior goals based on participants’ Apple watch health data. |
| Modification | No modifications were made during the study. |
| Fidelity | To secure the fidelity of the study, the counselor followed a standardized leader’s guide, audio-recorded the sessions, and created structured documentation on the REDCap. Actual proportion of the sessions completed was reported separately in Results section. |
